# Kinases of eIF2a Switch Translation of mRNA Subset during Neuronal Plasticity

**DOI:** 10.3390/ijms18102213

**Published:** 2017-10-22

**Authors:** Ekaterina Chesnokova, Natalia Bal, Peter Kolosov

**Affiliations:** Cellular Neurobiology of Learning Lab, Institute of Higher Nervous Activity and Neurophysiology, Russian Academy of Sciences, Moscow 117485, Russia; admin@ihna.ru

**Keywords:** eIF2, eIF2α kinases, 5′-UTR, uORFs, neuronal plasticity, PKMζ, cell stress, neuronal pathologies

## Abstract

Compared to other types of cells, neurons express the largest number of diverse mRNAs, including neuron-specific ones. This mRNA diversity is required for neuronal function, memory storage, maintenance and retrieval. Regulation of translation in neurons is very complicated and involves various proteins. Some proteins, implementing translational control in other cell types, are used by neurons for synaptic plasticity. In this review, we discuss the neuron-specific activity of four kinases: protein kinase R (PKR), PKR-like endoplasmic reticulum kinase (PERK), general control nonderepressible 2 kinase (GCN2), and heme-reguated eIF2α kinase (HRI), the substrate for which is α-subunit of eukaryotic initiation factor 2 (eIF2α). Phosphorylation of eIF2α is necessary for the cell during stress conditions, such as lack of amino acids, energy stress or viral infection. We propose that, during memory formation, neurons use some mechanisms similar to those involved in the cellular stress. The four eIF2α kinases regulate translation of certain mRNAs containing upstream open reading frames (uORFs). These mRNAs encode proteins involved in the processes of long-term potentiation (LTP) or long-term depression (LTD). The review examines some neuronal proteins for which translation regulation by eIF2 was suggested and checked experimentally. Of such proteins, we pay close attention to protein kinase Mζ, which is involved in memory storage and regulated at the translational level.

## 1. Neuron-Specific Translation Initiation Regulation and the Role of Kinases Phosphorylating α-Subunit of Eukaryotic Initiation Factor 2 (eIF2α) in This Process

### 1.1. Neuronal Plasticity and Translation

Activation of different membrane receptors affects local de novo protein synthesis and causes changes at the proteome level in postsynaptic terminals. Synthesis of new proteins in these terminals requires the presence of an autonomous translation apparatus that is almost independent of the processes going on in neuronal soma. Certain mRNAs, pre-transported from the nucleus and “conserved” for long-term storage, as well as ribosomes and translational factors, should be present in the dendrites to quickly start the translation of target proteins in synapses. Regulation of this local translation, as well as its correlation with somatic translation processes, may be quite complex. For example, it was shown that activation of neurons by the neurotrophic factor brain-derived neurotropic factor (BDNF) leads to an augmentation of translation in postsynaptic regions but suppresses protein translation in the cell body. At the same time, administration of *N*-methyl-d-aspartate (NMDA), a synthetic agonist of ionotropic glutamate receptors, to synaptoneurosomes, causes a selective increase in the synthesis of the α-subunit of kinase Ca^2+^/calmodulin-dependent protein kinase II (CaMKII) that contrasts with a simultaneous decrease in the total translation level. So, complex changes in translation, caused by activation of some membrane receptors, usually are at least bidirectional, and for many kinds of receptors such changes are still the issue for future studies [1]. However, a common feature of almost all mechanisms of translational regulation in neurons is the constitutive suppression of protein synthesis, which is alleviated only under strictly defined conditions, normally as a result of postsynaptic receptors activation. Dysfunction of these mechanisms, which causes excessive synthesis of neuronal proteins, often leads to pathologies, such as hyperexcitability of cells and epileptiform activity [2], or protein aggregation and neurodegeneration [3,4]. Suppression of local protein synthesis at rest allows the quick start of translation in the case of synaptic activation and depends on several factors. Arguably, the majority of translation regulation mechanisms are processes occurring at the initiation stage. One of the most important factors affecting initiation is the function of kinases. These kinases can phosphorylate translation factors and alter their activity, leading eventually to significant changes in the whole proteome of the cell.

### 1.2. Eukaryotic Initiation Factor 2 (eIF2) and Four Kinases of Its α-Subunit

**Figure 1:**
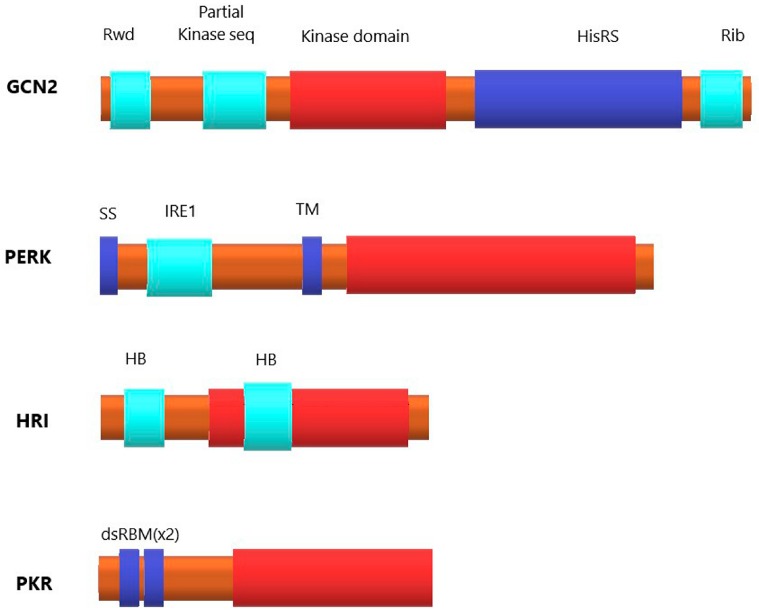
Domain structure of four kinases targeting α-subunit of eukaryotic initiation factor 2. The catalytic domain (red) is present in all four kinases. General control nonderepressible 2 (**GCN2**): the RWD sequence (domain in RING finger and WD repeat containing proteins and DEXDc-like helicases) associates with the activator protein GCN1 (general control of amino-acid synthesis 1-like protein 1), partial kinase domain is a cis-regulatory element, the HisRS (histidyl-tRNA synthetase)-related sequence binds with uncharged tRNA, and the C-terminal region facilitates GCN2 dimerization and its ribosome association. PKR-like endoplasmic reticulum kinase (**PERK**): the signal sequence (SS) facilitates translocation of N-part of kinase into the lumen of endoplasmic reticulum, the IRE1 (inositol-requiring enzyme 1)-related sequence is the unfolded protein response sensor, TM is the transmembrane segment. Heme-regulated eIF2α kinase (**HRI**): the molecule contains two heme-binding regions, one of which is inserted into the catalytic domain. Protein kinase R (**PKR**): two dsRBM motifs interact with double-stranded RNA.

Eukaryotic initiation factor 2 (eIF2) is arguably the most important initiation factor involved in neuron-specific translation regulation. It is the main part of the initiation ternary complex (eIF2-Met-tRNAi-GTP) and one of the key translation rate regulators in the cell [5]. Phosphorylation of the α-subunit of eIF2 arrests translation initiation. This phosphorylation can be accomplished by one of four kinases: PKR, HRI, GCN2 or PERK, all of which target Ser51 in eIF2α [6]. All these kinases have different domain structures, and the only domain common for all four is the catalytic domain (Figure 1). All eIF2α kinases are supposed to be activated by homodimerization and autophosphorylation [7,8,9]. Basically, GCN2 (general control nonderepressible 2) kinase is activated in response to amino acid deficiency, PERK (PKR-like endoplasmic reticulum kinase) responds to endoplasmic reticulum stress, HRI (heme-regulated eIF2α kinase) is activated by heme deprivation in erythroid cells, and PKR (protein kinase R) participates in an antiviral defense pathway involving interferon [7]. Interestingly, there is some data indicating that the function of eIF2α kinases is not limited to their kinase activity: PERK and PKR can also regulate the expression of specific proteins (such as cyclin D1 and tumor suppressor p53) by promoting their degradation in proteasomes [10].

#### 1.2.1. Kinase General Control Nonderepressible 2 (GCN2)

GCN2 is the primary responder to nutritional deprivation and the only eIF2α kinase conserved among virtually all eukaryotes [7]. GCN2 has the most complicated structure among four eIF2α kinases. Besides a typical eukaryotic kinase domain, it also harbors a pseudo-kinase domain and a histidyl-tRNA synthetase (HisRS)-related domain [7,9]. The mechanism of GCN2 activation during amino acid depletion involves the binding of uncharged tRNAs in the cytoplasm to the HisRS-related domain of GCN2. Notably, activation of GCN2 occurs not only as a result of histidine starvation, but after deficit of some other amino acids as well, and also by various disruptions of aminoacyl-tRNA synthesis or amino acids turnover [7]. Moreover, GCN2 can also be activated by such stresses as viral infection, proteasome inhibition and UV irradiation [9]. tRNA after binding to GCN2 triggers a conformational change of the kinase molecule that results in autophosphorylation and autoactivation of GCN2 [7]. Mice with knocked-out GCN2 show sensitivity to nutritional deficiencies and aberrant eating behaviors [11]. GCN2 is also the predominant eIF2α kinase expressed in the brain [12].

#### 1.2.2. PKR-like Endoplasmic Reticulum Kinase (PERK)

PERK is an endoplasmic reticulum (ER) transmembrane protein, and its regulatory region is situated in the lumen of the ER, while the eIF2α kinase domain is protruded into the cytoplasm. Accumulation of misfolded proteins inside ER, that may be caused by calcium dysregulation, oxidative damage, nutritional deficits among other reasons, results in the complicated combination of processes within ER known as unfolded protein response (UPR), a variant of ER stress. This response includes translation shift via PERK phosphorylation of eIF2α, which reduces the overall transcription rate and thus decreases the influx of nascent proteins into the ER, along with activating a program of gene expression designed to expand the processing capacity of the ER and to enhance ER-associated protein degradation. The UPR is important in the pathogenesis of many diseases, including diabetes, renal disorders, neuropathologies, and cancer. Misfolded proteins that accumulate in the ER lumen during ER stress are suggested to provoke the dissociation of PERK from the ER chaperone BiP/GPR78, thus leading to PERK oligomerization, which facilitates PERK autophosphorylation and autoactivation [7]. Intriguingly, a mechanism of PERK activation in the absence of misfolded proteins and without changes in the expression of ER chaperones after ischemia in the brain was described, suggesting more complex and nuanced control of PERK signaling, which may be mediated through changes in ATP or Ca^2+^ levels within the ER, which are in turn sensed by BiP or co-chaperone. Indeed, Ca^2+^ and calmodulin-dependent phosphatase calcineurin was identified as a direct modifier of PERK activity. In mice, deletion of PERK leads to neonatal insulin-dependent diabetes, epiphyseal dysplasia, hepatic and renal complications [11]. Loss-of-function mutation of PERK is the cause of Wolcott–Rallison syndrome in humans, a disease characterized by neonatal onset of diabetes, as well as defects in the pancreas and in the skeletal system [9]. In regard to the neuronal function of PERK, recent studies suppose that it may be one of the key proteins involved in memory impairments and neurodegeneration in Alzheimer’s disease. It was also implicated that PERK dysregulation is important in the pathogenesis of other neurodegenerative disorders, such as Parkinson’s disease, Huntington’s disease, amyotrophic lateral sclerosis and frontotemporal dementia [6]. Some data also indicate that alterations of normal PERK function are connected with aging-related deterioration processes in neurons, as was shown in the study of retinal cells of aged rats [13].

#### 1.2.3. Heme-regulated eIF2α kinase (HRI)

HRI plays an important role in erythroid cells. It contains two regions that can bind to heme, one at the N-terminal and the second within the kinase domain. Heme binding represses kinase activity, so a reduced amount of heme in erythroid cells triggers HRI activation [7]. Besides heme, both nitric oxide (NO) and carbon monoxide (CO) can bind to the N-terminal heme-binding domain of HRI [8]. NO acts as an activator of HRI [8,14,15], while CO serves as a suppressor of NO-induced HRI activation [8]. It was initially thought that HRI expression is limited to erythrocytes, but then it was demonstrated that it is also present in the liver and in macrophages [9]. Importantly, in the recent study, HRI was also found in neurons and confirmed to be important for memory consolidation [14].

#### 1.2.4. Protein Kinase R (PKR)

PKR is only conserved in vertebrates and it is not found in plants, fungi, protists, and invertebrates [8]. PKR was initially discovered as a kinase that phosphorylates eIF2α in response to viral infection, thereby blocking the translation of viral mRNAs and promoting apoptosis in response to viral infection. However, PKR can also be activated in response to other diverse signals, such as oxidative and ER stress, cytokine signaling and growth factors. This kinase has been implicated in the pathogenesis of obesity and cancer. The structure and the mechanism of activation of PKR are the best characterized among four eIF2α kinases. PKR is localized in the cytosol and the nucleus. Its regulatory N-terminal region contains a double-stranded RNA binding domain that inhibits the catalytic domain in the absence of ds-RNA. The C-terminal catalytic domain has a dimerization site. Another important regulator of PKR besides dsRNA is PACT (Protein Activator), which becomes phosphorylated in response to various stress factors. PKR has many targets besides eIF2α. It phosphorylates p53, indirectly facilitates activation of the STAT transcription factors, promotes MAPK activity, induces expression of the pro-apoptotic transcription factor CHOP (CCAAT-Enhancer-binding protein Homologous Protein) and mediates NF-κB activation. In general, the role of PKR in response to ER stress seems to be in direct opposition to that of PERK: activation of PKR eventually leads to apoptosis (but not always) [9].

### 1.3. The Mechanism of Translation Shift Caused by eIF2α Phosphorylation and Its Role in Neurons

Activation of kinases phosphorylating eIF2α suppresses general translation, but also selectively stimulates the translation of some specific mRNAs. Typically, these mRNAs have long 5′-UTR with complicated secondary structure and a few short upstream open reading frames (uORFs) providing multiple “false starts” of translation initiation and thus hampering the initiation on the main frame. All these features mean that initiation of translation from such mRNAs is consistently low, the rate-limiting stage of their translation is not initiation but elongation, and so they thrive when eIF2α is phosphorylated and initiation of other mRNAs is suppressed [16]. Most of such proteins participate in cell stress response, and particularly in ER stress [17,18,19,20,21,22,23].

Global effects of eIF2α phosphorylation during cell stress are, first, conservation of energetic resources achieved by decrement of total translation and, second, outbreak of proteins necessary for the repair of stress-caused damage—or for induction of apoptosis. Some of these proteins are regulated transcriptionally, while p-eIF2α controls the level of correspondent transcription factors. Thus, eIF2α phosphorylation results in increased translation of transcription factors ATF4, ATF5, ATF6, XBP-1 and CHOP which, in turn, selectively induce the transcription of multiple genes coding for ER chaperones and enzymes needed to cope with the excess of unfolded proteins in the ER or trigger apoptosis. Other proteins important for overcoming cell stress, such as VEGF-A (angiogenic factor that is important for compensation of chronic hypoxia effects) or GADD34 (necessary for p-eIF2α dephosphorylation and reversal of stress-caused changes) are believed to be upregulated by p-eIF2α directly at the translational level [23].

However, there is much data indicating that in neurons, p-eIF2α-driven translational shift is a necessary part of normal neuronal function in the absence of stress. eIF2 is an important regulator of synaptic plasticity (noteworthy, some of p-eIF2α downstream proteins are important for both stress response and neuronal plasticity) [23,24,25,26,27,28,29].

Considering the dual role of p-eIF2α in neuronal cells, we will discuss below its role in both stressed and normally functioning neurons, but will focus on the latter. It must also be noted that in some works reporting some aspects of eIF2 role in synaptic plasticity and memory, the methods used were in fact in vitro or in vivo models of some pathological conditions [30,31], so the neurons examined in these works most likely endured cellular stress, and it is hard to discriminate the role of eIF2 in stress signaling from its neuron-specific function by analyzing these results (one paper [32] is a rare example of work in which the authors actually checked if the changes they observed in neurons were caused by cellular stress).

## 2. Neuron-Specific Proteins that May Be Regulated by eIF2α Phosphorylation

The list of proteins that have three things in common—first, it is known that their mRNAs contain uORFs in their 5′-UTR; second, expression of their mRNAs in neurons was established; and third, regulation of their translation in neurons via eIF2-related mechanisms was proposed—is presented in Table 1.

### 2.1. Activating Transcription Factor 4 (ATF4)

Presumably the most important protein that is known to be regulated translationally by phosphorylation of eIF2α and associated both with cell stress and neuronal activity is activating transcription factor 4 (ATF4), also known as cAMP-response element binding protein 2 (CREB-2) [12,18,19,20,38]. ATF4, despite its name, is a highly conserved repressor of CREB-mediated gene expression, and ATF4-mediated transcription block must be released for the late phase of long-term potentiation (L-LTP) and formation of long-term memory [39,40].

The 5′-UTR of ATF4 mRNA contains two uORFs, conserved from invertebrates to mammals [41]. The first uORF is very short, and most of scanning ribosomes reinitiate at uORF2 after termination of uORF1 translation. The second uORF overlaps with the first 83 nucleotides of the main reading frame. If there are many ternary complexes available, a large fraction of scanning ribosomes initiate on the uORF2, thus terminating downstream of the ATF4 major initiation codon. Ribosomes cannot scan backwards, so translation from the main reading frame is low. In contrast, when eIF2α is phosphorylated, the number of ternary complexes is reduced, and many of the scanning 40S subunits cannot initiate on the second uORF, thus bypassing it, sliding further, and continue scanning, which makes translation initiation at the major ATF4 start codon more likely [27].

In research by Jiang et al. [29], a conditional transgenic mouse strain was generated, in which PKR-mediated phosphorylation of eIF2α was specifically increased in hippocampal CA1 pyramidal cells by the chemical inducer. Administration of this inducer significantly increased translation of ATF4 (with no obvious reduction in general translation), and this led to suppression of CREB-dependent pathways in CA1 cells, impairment of hippocampal late-phase LTP and memory consolidation, as was shown in electrophysiological experiments with hippocampal slices and in behavioral experiments with contextual fear conditioning. At the same time, inhibition of general translation by low-dose anisomycin did not block hippocampal-dependent memory consolidation [29].

Despite the established role of ATF4 as a suppressor of synaptic plasticity, it cannot be said that a constitutive decrease of ATF4 concentration in neurons enhances L-LTP and promotes memory formation, since the changes are more complicated and, in some respect, bidirectional, as was shown in experiments with GCN2^−/−^ mice. These mice have a decreased eIF2α phosphorylation level and decreased concentration of ATF4 in their hippocampal neurons, as was demonstrated by Western blotting. In hippocampal slices from these mice, a single 100-Hz train in CA1, that is not enough for L-LTP formation in wild-type mice, induced a strong and sustained L-LTP. In contrast, electrical or chemical stimulation variants that normally elicit L-LTP in wild-type slices, failed to evoke L-LTP in GCN2^−/−^ slices. Somewhat similar results were obtained in behavioral tests in vivo (Morris water maze): after weak training (once a day), the spatial memory of mutant mice was improved compared to wild-type control, but it was impaired after more intense training (three times a day). In some sense, the absence of ATF4 block makes neurons over-sensitive to stimulation, and their potentiation occurs “too easy”. So, neuronal activity-dependent shifts in ATF4 level, caused by GCN2 activity, are important for long-term potentiation to occur normally [12].

In another work concerning ATF4, APP/PS1 mice (double transgenic mice expressing a chimeric mouse/human amyloid precursor protein and a mutant human presenilin 1 in their neurons), an established animal model of Alzheimer’s disease, was used. First, the authors confirmed the increase of ATF4 translation and the elevated eIF2α phosphorylation level in hippocampi of APP/PS1 mice (compared to wild-type mice) by Western blotting. Next, using the Cre-loxP technique, additionally genetically modified mice were bred, APP/PS1, in which PERK was conditionally removed in excitatory neurons in the forebrain and hippocampus late in development. The genetic deletion of PERK prevented enhanced eIF2α phosphorylation, caused an increase in the overall translation level and selective suppression of ATF4, and rescued impairment of synaptic plasticity (high-frequency stimulation of acute hippocampal slices was used to assess LTP formation) and spatial memory (estimated by Morris water maze, Y water maze and object location test), in these mice. Mice from another mutant line, APP/PS1 with constitutive deletion of GCN2, also demonstrated improved synaptic plasticity and spatial learning compared to APP/PS1, but GCN2 knockout did not decrease the basal eIF2α phosphorylation level as drastically as PERK knockout (in APP/PS1 mice with PERK knockout, eIF2α phosphorylation was even lower than in wild-type animals). So, the authors supposed that PERK may be more important for eIF2 regulation than GCN2 [31].

### 2.2. ER Stress-Related Proteins: Growth Arrest and DNA Damage-Inducible Protein (GADD34) and CCAAT-Enhancer-Binding Protein Homologous Protein (CHOP)

CHOP and GADD34 proteins participate in the same signaling pathways as ATF4 and, being structurally unrelated, have much in common in their regulation and function. Both CHOP and GADD34 are connected with ER stress response. The expression of both these proteins is regulated by ATF4 [20,38], and it was also demonstrated that CHOP can directly activate GADD34 [42].

In the work by Biever et al. [32], mice were repeatedly administered with D-amphetamine (systemic injection), and then overall translation changes in their striatum were observed by polysome profile analysis on striatal lysates, and differential expression of selected genes was assessed by RT-qPCR. In this particular work, direct molecular targets of amphetamine in neurons were not discussed, but it is well known that this drug increases the concentration of catecholamines in the synaptic cleft by several distinct mechanisms (see [43] for review). Polysome profiling of striata of amphetamine-treated mice showed an increase in the amplitude of the “vacant” 80S monosome peak along with a reduction in the polysome population (compared to saline-treated mice). It means that amphetamine causes a decrease of global mRNA translation in the striatum. This result was confirmed in another experiment, in which lysates were treated with puromycin, an antibiotic that binds selectively to nascent protein chains and allows to measure de novo protein synthesis level. A puromycin-based assay demonstrated that the overall protein synthesis level in striatal ribosomes was decreased in amphetamine-treated mice. Using Western blotting analysis of whole striatal lysates at different time points, the authors also confirmed a robust enhancement of phosphorylated eIF2α, most prominent at 30 min after amphetamine injection. The targets selected for RT-qPCR were several mRNAs known to contain uORFs in their 5′-UTR. Two studied uORF-bearing mRNAs, GADD34 and CHOP, were found to be enriched in polysomal fractions of amphetamine-treated mice, indicating that the translation of these mRNAs was upregulated. (Unexpectedly, ATF4 translation was unchanged after amphetamine administration.) While GADD34 mRNA was also enriched in non-polysomal fractions, suggesting that GADD34 was enhanced not only translationally but also transcriptionally, CHOP mRNA was only enriched in polysomal fractions.

GADD34 (growth arrest and DNA damage-inducible protein) is also known as PPP1R15A (protein phosphatase regulatory subunit 15A). It plays an important role in recovery from endoplasmatic reticulum stress because it dephosphorylates p-eIF2α and thus reactivates normal protein synthesis. In neurons, the function of this protein is associated with the recovery from ischemia [44]. Considering that GADD34 is both a protein activated by p-eIF2α and a necessary factor for p-eIF2α dephosphorylation, it provides a negative feedback loop, preventing uncontrollable p-eIF2α accumulation and stress-related decompensation processes in the cell. It should also be noticed that proper GADD34 function requires the presence of monomeric G-actin [45,46]. Mouse GADD34 mRNA contains two overlapping and out of frame uORFs [47,48]. CHOP (CCAAT-enhancer-binding protein homologous protein) is also known as DDIT3 (DNA damage-inducible transcript 3), and is a transcription inhibitor important for ER stress response and apoptosis induction. CHOP apparently has no specific role in normally functioning neurons, as it is known to be upregulated in neurons only in different stress conditions [49,50]. CHOP-mediated apoptosis may be involved in the development of Parkinson’s disease, polyglutamine disease [51] and amyotrophic lateral sclerosis [52]. The 5′-UTR of human CHOP mRNA contains three uORFs, but only the second one of them is not in-frame with the leader sequence and is highly conserved between human, mouse and hamster. Experiments with genetic constructs containing wild-type or mutated CHOP mRNA 5′-UTR and luciferase reporter showed that this uORF inhibits translation from the main reading frame. Further changes in this construct, including different mutations that had a small effect on mRNA structure but drastically changed the sequence of the uORF-encoded short peptide, demonstrated that this peptide itself is partly responsible for the repression of main frame transcription. In HeLa cells expressing constructs with upstream start codon in place, but peptide-altering mutation within the upstream reading frame, the level of reporter was higher than in cells expressing constructs with wild-type 5′-UTR but still lower than in cells expressing constructs with 5′-UTR without upstream start codon [33].

Considering that both proteins that were shown to be translationally up-regulated by systemic amphetamine administration are important for ER stress response, the authors specifically tested if an eIF2α-mediated translation shift in neurons was caused by cellular stress mechanisms. They measured the levels of some proteins that are known as markers for ER stress (this list included PERK), and also markers of apoptosis and gliosis, in striatal lysates of experimental animals. It was shown that striatal levels of almost all evaluated markers were not altered by amphetamine, indicating that amphetamine exposure was not accompanied by ER stress or neurotoxicity. It allowed the authors to conclude that the regulation of translation by D-amphetamine most likely contributes to persistent modifications altering striatal plasticity rather than representing a protective mechanism to cope with an insult [32]. Biever et al. registered eIF2α phosphorylation increase correlating with GADD34 and CHOP expression increase, but did not identify which of the four eIF2α kinases was responsible for this effect. However, in another work, it was demonstrated that cell stress-dependent translation of GADD34 is partially suppressed in *PERK^−/−^* cultured CHO-K1 cells and almost completely suppressed in *GCN2^−/−^* cells [20], so supposedly both these kinases participate in GADD34 regulation; GCN2 is more important for this, and there still may exist some alternative pathways for GADD34 expression activation bypassing both these kinases (and maybe even completely independent of eIF2α phosphorylation). This third proposal is confirmed, for example, by the fact that in specific conditions (viral infection), GADD34 in fibroblasts is activated via PKR kinase [21].

As for CHOP, in the described above work with an Alzheimer’s disease animal model, CHOP concentration was found to be unaltered in APP/PS1 mice with conditional PERK knock-out compared to APP/PS1 mice [31], so we may speculate that PERK is not important for CHOP translation regulation. Consistent with this, CHOP upregulation after chemically induced stress in mouse embryonic fibroblasts was proved to be caused by GCN2 [22].

### 2.3. Beta-Site APP-Cleaving Enzyme 1 (BACE1), the Enzyme Connected with Alzheimer’s Disease

The role of eIF2 in Alzheimer’s disease was already mentioned above in regard to ATF4 translation regulation [31]. However, ATF4 is not the only protein for which translation is regulated by an eIF2-dependent mechanism that becomes disrupted in Alzheimer’s disease. Beta-site APP-cleaving enzyme 1 (BACE1) is the rate-limiting enzyme for β-amyloid production. BACE1 concentration increase is typical for Alzheimer’s disease. It is known that BACE1 mRNA 5′-UTR is a cis-acting translational repressor because it is long, GC-rich, has a complicated secondary structure, and contains four uORFs [14].

In the research addressing eIF2-mediated translation regulation of BACE1, glucose starvation was used as a model of cell stress [30]. There is some data indicating that BACE1 increase in Alzheimer’s disease might be a result of translational derepression of the BACE1 mRNA 5′-UTR induced by energy metabolism stress. It was shown that, in primary neuronal cultures, glucose deprivation causes BACE1 protein level increase by translational mechanism (Western blotting assay showed a boost of BACE1 protein concentration, and BACE1 mRNA concentration, estimated by RT-qPCR, was not increased). The eIF2α phosphorylation level was also increased in these cultures. Conforming to this, in in vivo experiments, chronic energy deficit, induced by treatment with energy metabolism inhibitors, caused an increase in eIF2α phosphorylation, BACE1 level and amyloidogenesis in Tg2576 mice (these mice overexpress human amyloid precursor protein).

To specify which of the eIF2α kinases is involved with BACE1 level regulation, the authors transiently transfected BACE1-293 cells (HEK-293 stably overexpressing BACE1) with constructs encoding dominant-negative PERK (PERKDN) or GCN2 (GCN2DN), and on the next day treated the cells for 12 h in media with or without glucose. It was shown that glucose-deprivation-induced increases in BACE1 levels were completely prevented by overexpression of PERKDN, but not GCN2DN allele, demonstrating that PERK was the kinase responsible for eIF2α phosphorylation and the BACE1 increase, and that GCN2 kinase was not involved in this mechanism. To further confirm the role of PERK, a few experiments, in which eIF2α phosphorylation by PERK was prevented, were performed: cell cultures were transfected with either constitutively active p-eIF2α phosphatase PP1c regulatory subunit, or with dominant-negative PERK, or with PERK inhibitor P58IPK. In all three cases, in transfected cells, energy deprivation-induced BACE1 increase was blocked, confirming once again that activation of PERK is necessary for the energy-deprivation-induced BACE1 increase. The authors did not address PKR or HRI kinases, because according to literary data, these two kinases are not activated during nutritional deficits [30]. However, in another work, it was demonstrated that HRI kinase is also important in BACE1 translation regulation. Mouse synaptosomes were activated with glutamate, which caused a significant increase in BACE1 and phosphorylated eIF2α concentrations, as was shown by Western blotting. It was demonstrated before that NO synthesis is a part of the signaling cascade triggered by NMDA receptor activation, so the authors addressed possible NO participation in glutamate-induced BACE1 synthesis boost. Co-treatment with glutamate and 7-NI (NO-synthase inhibitor) did not cause the increase in BACE1 concentration, suggesting that glutamate-induced eIF2α phosphorylation and BACE1 translation are NO-dependent. This was further confirmed by treating synaptosomes with NO donor SNP, after which BACE1 concentration increased. The same results were obtained with primary cultures of cortical neurons. Next, the authors demonstrated that HRI mRNA and protein are present in human hippocampus, mouse hippocampal neurons and human neuroblastoma cells. Using immunostaining, they also found that HRI is colocalized with the postsynaptic marker PSD95 in the synaptic spines as well as p-eIF2α. To study the role of HRI in BACE1 expression in human neuroblastoma SH-SY5Y cells, two methods were used: a specific HRI inhibitor administration and a transfection with small interfering RNA causing knockdown of HRI expression. Cells pre-incubated with the HRI inhibitor failed to respond to SNP treatment and did not overexpress BACE1 nor increase the levels of phosphorylated eIF2α. Similarly, when HRI expression was knocked down by a specific siRNA, SNP did not up-regulate BACE1 protein. Consistent with these in vitro findings, NOS inhibitor or HRI inhibitor, being administered chronically to mice for one week, induced a significant reduction in the levels of BACE1 and p-eIF2α in the synaptosomes isolated from the hippocampi of these mice. The authors also demonstrated that the administration of SNP to cells induced spine growth, that was prevented by pre-incubating the cells with the HRI inhibitor, but not with the PKR inhibitor. In experiments in vivo, two paradigms involving hippocampal processing were used: the object-recognition test and the context-recognition test. Inhibitors of HRI or NOS were administered intraperitoneally to mice during the memory consolidation period. Mice that received each of these inhibitors showed a memory impairment in the object-recognition without affecting their exploratory behavior. Similarly, context-recognition memory was significantly compromised by both inhibitors. These data confirm that HRI is essential for BACE1 expression regulation and memory consolidation in mice [14].

### 2.4. Glutamate Ionotropic Receptor NMDA Type Subunit 2B (GluN2B)

Glutamate receptors play a key role in nervous system function, particularly in memory and learning. One of these receptors, *N*-Methyl-d-aspartate receptor (NMDAR), is crucial for synaptic plasticity and participates in both long-term potentiation (LTP) and long-term depression (LTD) [53,54], but its role in LTP is more well-known. This receptor is a heterotetrameric cation channel. While it always consists of four subunits, there are several possible types of these subunits, and combinations of them are different in different NMDAR receptors. Subunit GluN2B is considered to be important for brain development and also for mature neuronal function. GluN2B interacts directly with PSD-95 scaffold protein, and this interaction is the first step in a few intracellular signal cascades caused by NMDAR activation. As was shown in experiments with primary neuronal cultures, glutamatergic stimulation causes de-repression of GluN2B translation, providing rapid protein accumulation necessary for neuronal activity, spine growth and memory formation. It was shown that the observed translation shift is regulated by eIF2α kinase HRI, as the effect was avoided when a specific HRI inhibitor or a small interfering RNA that binds with HRI mRNA (siHRI) were used. GluN2B translational de-repression was also dependent on Ca^2+^ signaling and mediated by neuronal NO-synthase, since treatment with either Ca^2+^ chelator or nNOS inhibitor prevented the glutamate-induced increase in GluN2B expression. Similar results were obtained in cortical synaptosomes and in isolated postsynaptic membranes. The authors proposed that HRI is activated following the interaction of NO with its heme sensor, inducing a transient phosphorylation of eIF2α. GluN2B mRNA has a long 5′-UTR with three uORFs. Human neuroblastoma cells, transfected with the wild type GluN2B 5′-UTR-containing construct, showed reduced luciferase reporter expression compared to control cells (transfected with the vector without this 5′-UTR). Treatment with NO donor SNP caused a significant increase in reporter expression in these cells. Mutation of all three upstream AUGs reversed 5′-UTR-dependent basal translation repression, but in this case SNP did not cause changes in reporter expression level, so GluN2B mRNA lacking these three uORFs is unable to shift its translation rate after glutamate receptor activation [15].

### 2.5. Oligophrenin-1

The paper by Di Prisco et al. [28] is also dedicated to glutamate signaling. These authors focused on another type of glutamate receptors: group I mGluRs. These receptors are metabotropic and bear no structural resemblance to NMDAR, but like NMDAR, mGluRs are also very important for synaptic plasticity and may facilitate both LTP and LTD [55]. However, currently there is more data about mGluR-dependent LTD. Unlike NMDAR-dependent LTD, which persists after treatment with translation inhibitors, the mGluR-LTD mechanism requires rapid protein synthesis within minutes after receptor activation [56]. The shift in translation during mGluR-LTD resembles some processes typical for LTP: for mGluR-LTD, eIF2α phosphorylation is also crucial, and there is a decrease in overall translation paired with the intensification of translation of specific mRNAs. One of the main results of mGluR-LTD that is observed at the cell proteome level is a diminishing of surface AMPA-receptors density at synapses. Di Prisco et al. identified eIF2 as a master effector of mGluR-LTD-dependent translation changes using a few different experimental approaches.

First, they activated mGluR in mouse hippocampal slices with dihydroxyphenylglycine (DHPG, a selective mGluR1/5 agonist) and demonstrated that DHPG causes mGluR-LTD and consistently increases eIF2α phosphorylation in the slices. Sal003, a selective inhibitor that blocks p-eIF2α phosphatases, had the same effects, but Sal003-induced LTD was also insensitive to mGluR1 and mGluR5 antagonists, indicating that the increase in eIF2α phosphorylation may be a downstream effect of mGluR activation.

To test whether eIF2α phosphorylation is required for mGluR-LTD at CA1 synapses, the authors used *Eif2s1^S/A^* heterozygous knock-in mice, containing one mutant eIF2α-encoding allele (in mutant protein, the phosphorylation site is absent). These mice have reduced background eIF2α phosphorylation in the hippocampus. In experiments with acute hippocampal slices, DHPG or paired-pulse low frequency stimulation caused mGluR-LTD in wild-type, but not *Eif2s1^S/A^* slices. Still, NMDAR-LTD, elicited by low frequency stimulation, occurred normally in *Eif2s1^S/A^* slices, indicating that eIF2α phosphorylation is necessary only for the protein synthesis-dependent variant of LTD. Another transgenic mouse line (fPKR) was used to investigate the effect of opposite change—overly increased eIF2α phosphorylation—on LTD formation in CA1 neurons. Using the Cre-loxP recombination technique, the authors modified the PKR gene in some neurons to make PKR catalytic activity inducible by a specific chemical agent, and simultaneously tagged these neurons with green fluorescent protein (GFP). In paired recordings from GFP− and GFP+ neurons, the PKR activity-inducing agent generated a sustained LTD only in GFP+ neurons, so the upregulation of PKR activity alone is enough for LTD induction. Taken together, these data indicate that PKR kinase is necessary for mGluR-LTD-caused eIF2α phosphorylation. Supposedly, GCN2 may be also important for this process: these two kinases are synergic in suppression of long-term potentiation, as was demonstrated previously in experiments with hippocampal slices [12,26,57].

To further explore translation changes paired with mGluR-LTD, RNA-seq was used to analyze total mRNA abundance, mRNAs that are poorly or not translated (monosome fraction), and the actively translating mRNAs (polysome fraction) in control and DHPG-treated cultured mouse neurons. The authors identified mRNAs for which translation was induced by DHPG and checked if there are uORFs in their 5′-UTRs. Altogether, 324 different mRNAs for which translation was upregulated by mGluR activation were discovered, 72 of them containing uORFs. This list included subunits of membrane receptors, ion channels, translation factors, kinases and other enzymes participating in intracellular signaling pathways (the full list is available in supplementary data to the discussed article). However, upregulation of oligophrenin-1 (Ophn1) mRNA translation caused by mGluR-LTD became the most important result of this screening. Oligophrenin-1 is a Rho-GTPase-activating protein which is necessary for intracellular signal transduction and regulation of actin cytoskeleton restructuring. It is important for proper spine formation, activity-dependent maturation and plasticity of excitatory synapses, maintaining their structural and functional stability. Mutation of oligophrenin-1 has been associated with X-linked mental retardation, a hereditary condition with some characteristic abnormalities in brain morphology [58]. Oligophrenin-1 mRNA harbors two uORFs in its 5′-UTR. To test whether these uORFs are cis-acting regulators of the translation rate, a genetic construct, in which the 5′-UTR of Ophn1 was fused to the coding region of the luciferase reporter, was transfected to HEK-293T cells. The expression of this construct was reduced compared to the control vector without Ophn1 mRNA 5′-UTR. In cells treated with thapsigargin (a toxin that causes cellular stress and eIF2α phosphorylation), translation of the Ophn1 mRNA 5′-UTR-containing construct was upregulated, which confirms that uORFs are necessary for switching the translation level of Ophn1 [28].

It was demonstrated before that hippocampal LTD is crucial for object-place recognition, so to test LTD-dependent changes in vivo, Di Prisco et al. chose the spatial recognition task, in which the same objects are presented twice in two days. ISRIB (an agent that prevents the translational effects of eIF2α phosphorylation) administered to mice before training caused an increase in object exploration time at the reexposure day (notably, ISRIB had no effect on distances traveled or exploration times during training). Conforming to that, mutant *Eif2s1^S/A^* mice also spent more time exploring the objects during reexposure compared to the wild-type mice. Both these findings show that eIF2-mediated translational control is needed to successfully learn the novel object-space configuration. Also, Ophn1 shRNA-injected mice spent significantly more time exploring the objects during reexposure compared to control mice (injected with scrambled shRNA). So, behavioral experiments also confirmed the importance of oligophrenin-1 for LTD formation and correlation between oligophrenin-1 function and eIF2α phosphorylation [28]. At the same time, in the abovementioned study by Biever et al. with amphetamine-treated animals, Ophn1 mRNA was not enriched in polysomal fractions, and the oligophrenin-1 protein level in lysates remained unchanged, suggesting that oligophrenin-1 is not important for catecholamine-induced neuronal activation [32].

### 2.6. Postsynaptic Density Proteins: Synapse-Associated Protein 90/Postsynaptic Density Protein-95-Associated Protein 3 (SAPAP3) and SH3 and Multiple Ankyrin Repeat Domains 1 (Shank1)

In postsynaptic density, SAPAP3, Shanks and PSD-95 are the three master scaffolding proteins that cross-link neurotransmitter receptors, signaling molecules and cytoskeletal components [34].

SAPAP3 stands for synapse-associated protein 90/postsynaptic density protein-95-associated protein 3 (also known as DLGAP3, DLG associated protein 3). Mutations of SAPAP3 were shown to be important for pathogenesis of obsessive-compulsive disorder [59,60]. SAPAP3 mRNA is long, GC-rich and contains four uORFs, but only uORF2 overlaps with the main frame: in the sequence AUGA, the last two nucleotides of the SAPAP3 start codon AUG are simultaneously the first two nucleotides of the uORF2 stop codon UGA. Using molecular constructs with AUG substituted to AAG in different uORFs, it was confirmed that only the second uORF strongly down-regulates translation efficiency. Ribosomes translating uORF2 bypass AUG^+1^ and are thus unable to synthesize full-length SAPAP3 [34]. Moreover, the alternative translation initiation site within the same protein-coding frame (AUG^+277^) is present in SAPAP3 mRNA, and so two distinct SAPAP3 protein isoforms may be produced. It was shown that concentrations of these two isoforms vary in different rodent brain regions: SAPAP3β (truncated isoform) is the predominant isoform in the hippocampus, thalamus, cerebellum and brain stem, while in olfactory bulb, SAPAP3α (the long isoform) prevails, and in the neocortex, both isoforms are presented equally. In an experiment with transfected HEK293 cells, point mutation of uORF2 in SAPAP3 mRNA shifted the isoforms’ ratio in favor of SAPAP3α, so uORF2 is not only necessary for mRNA translation inhibition, but also may be important for isoforms’ proportion regulation. It may be speculated that ribosomes translating uORF2 bypass AUG^+1^, stop translation shortly thereafter and may subsequently reinitiate translation at AUG^+277^ leading to SAPAP3β synthesis (noteworthy, the first 300 nt of the SAPAP3 ORF are highly conserved in various vertebrate species). To assess whether eIF2α phosphorylation may affect the relative initiation rates occurring at AUG^+1^ and AUG^+277^ of SAPAP3 mRNA in vivo, SAPAP3α:SAPAP3β ratios were calculated in the neocortex, hippocampus and cerebellum of heterozygous eIF2α^+/S51A^ knock-in mice possessing reduced p-eIF2α levels. Unexpectedly, isoform ratios were found to be unaltered in mutant mice compared to wild-type mice, suggesting that while uORF2 is a cis-regulating element of SAPAP3 mRNA, eIF2 may not be important for its control and it is regulated by some alternative ways. In this work, no experiments with eIF2α kinases were performed [34].

Shank1 (SH3 and multiple ankyrin repeat domains 1) also has complicated translational regulation. Shank family proteins regulate the morphology of dendritic spines, and Shank1 mRNA is abundant in dendrites of hippocampal neurons and cerebellar Purkinje cells. In humans, mutations in genes coding Shank proteins have been associated with mental retardation and autism. It was shown that translation of human Shank1 mRNA is inhibited by its own 5′-UTR. The organization of this 5′-UTR is very complex. To begin with, it has high GC content and forms an intricate secondary structure. Also, this 5′-UTR contains two alternative translation initiation sites within the same protein-coding frame (AUG^+1^ and AUG^+214^, producing two protein isoforms), three “normal” uORFs starting with AUG (from which uORF3 is the most important because it overlaps with AUG^+1^), and, most unusually, another uORF starting with the non-canonical ACG start codon (and overlapping with uORF3). To analyze the role of each uORF for translation efficiency, uORFs were individually disabled by point mutations within the context of luciferase reporter vectors, and the translation rate of fused mRNAs was assayed in a cell-free system (rabbit reticulocyte lysate), in HEK cells or in cortical neurons. Mutations of uORFs 1 or 2 did not alter the reporter translation rate, and mutation of uORF3 increased it, as was expected. However, mutation of the ACG start site caused almost complete loss of translation initiation at AUG^+1^, that was partially restored by additional uORF3 mutation. So, it was demonstrated that the extraordinary ACG uORF does not inhibit main frame translation but, on the contrary, maintains it, presumably by competing with inhibiting uORF3 for ribosomes and translation factors. Ribosomes that started translation at ACG uORF would bypass uORF3 and are therefore capable of reinitiating translation at AUG^+1^ to synthesize the full-length isoform of Shank1. Further evidence of the exceptional properties of Shank1 mRNA 5′-UTR is that cellular stress did not change the translation rate of reporter mRNAs fused to this 5′-UTR, indicating that eIF2α phosphorylation does not influence Shank1 translation. So, despite some similarities with p-eIF2α-dependent mRNAs, Shank1 mRNA is regulated by its own unique mechanisms [35].

### 2.7. Neuron-Specific BCL2-Antagonist/Killer (N-Bak), a Constitutively Repressed Pro-Apoptotic Protein

Bak (BCL2-antagonist/killer) is a pro-apoptotic factor. When activated, Bak and related protein Bax generate pores in the mitochondrial membrane, causing mitochondrial proteins to be released into cytosol to advance apoptosis. Unlike other types of cells, neurons do not express a classical form of Bak mRNA, because in neurons premature Bak mRNA is entirely spliced. The protein that may be translated from this spliced mRNA is called N-Bak. This protein is able to be translated and can be detected by specific antibodies, as was demonstrated in experiments with an in vitro translation system or transiently transfected HeLa cells [61]. Artifical overexpression of N-Bak induced apoptosis in cortical, hippocampal and cerebellar neurons but had an unexpected anti-apoptotic effect in sympathetic neurons [62].

N-Bak mRNA is present and stable in the brain and in cultured primary neurons and stored in granular structures, as was shown by in situ hybridization [36]. Despite the presence of its mRNA, N-Bak protein is not detectable in the same neurons. Presumably, an earlier report in which N-Bak was revealed in neurons by Western blotting [62] was erroneous, since in later studies it was established that the band that is occasionally recognized by anti-Bak antibodies and has expected molecular weight is actually non-specific [36]. Experiments with proteasome inhibitors demonstrated that the absence of N-Bak protein is not caused by its rapid proteasome-mediated degradation [61]. Instead, the N-Bak mRNA appears to be under a strong translational block.

5′-UTR is the same in both Bak and N-Bak transcripts. This 5′-UTR is not well conserved, and may contain one or two uORFs in different species. Jakobson et al. worked with mouse neurons and chose to address the first of two mouse uORFs, because it is in the context of the Kozak consensus sequence. Genetic constructs containing luciferase reporter downstream of wild-type or mutated N-Bak 5′-UTR (in mutated form, 38 nucleotides encompassing AUG of the first uORF were deleted) were made and transfected to mouse neuroblastoma Neuro-2a cells. The deletion increased luciferase activity, suggesting that uORF1 is important for translation repression. However, in this case, uORF is not the only mechanism of translation arrest: 3′-UTR of N-Bak mRNA also contributes to this, since it contains premature termination codon and exon–exon junction that are known to be necessary for nonsense-mediated translation repression (see [63] for the exact mechanism of this). In experiments with constructs containing wild-type or mutated 3′-UTR, the authors confirmed that these 3′-elements indeed diminish the reporter translation.

Jakobson et al. supposed that, like many mRNAs with uORFs, N-Bak mRNA translation may be “turned on” during cell stress by eIF2α phosphorylation; considering that N-Bak must be an apoptotic factor, they also performed an experiment with induction of apoptosis. Cellular stress was induced by thapsigargin; it was shown that the used dose of thapsigargin induced the maximal level of eIF2α phosphorylation. To induce mitochondrial apoptosis, cultured superior cervical ganglion neurons were deprived of nerve growth factor, and cortical neurons were treated with etoposide (an inhibitor of topoisomerase II) in the presence of caspase inhibitors. However, unexpectedly, endogenous N-Bak mRNA was still not translated in neurons during cellular stress, or even in apoptotic neurons, as was shown by antibodies and additionally confirmed with quantitative mass spectrometry analysis. Moreover, the dense N-Bak-mRNA-containing granules were still clearly visible in stressed or apoptotic neurons. It means that this protein does not participate in the classical apoptosis or stress response in neurons. Whether the N-Bak mRNA is ever released from the translational block is still unclear [36], but it may be speculated that N-Bak translation is perhaps permitted only in particular cases of apoptosis induced by some specific factors.

### 2.8. Protein Kinase Mζ, “the Memory Molecule”

One of the important neuronal proteins regulated by uORFs is an enzyme necessary for synaptic plasticity: atypical protein kinase Mζ (PKMζ). We are particularly interested in this kinase, so we decided to describe its regulation and functions more minutely and devote the whole next section to it.

## 3. Protein Kinase Mζ, Its Functions and Regulation of Its Translation

### 3.1. Protein Kinase Mζ Structure and Its Difference from Structures of Other Related Kinases

In the group of PKC Ser/Thr protein kinases, there are three subfamilies: conventional, novel and atypical PKCs (aPKCs). aPKC isoenzymes PKCζ and PKCι/λ have a functional role in insulin signaling, and their dysfunction correlates with insulin-resistance disorders. aPKCs, in contrast to other PKC subfamilies, are not regulated by diacylglycerol and Ca^2+^, because aPKCs lack the calcium-sensitive C2 domain and have a dysfunctional DAG-sensitive C1 domain [64,65]. Despite their participation in insulin-controlled pathways, aPKCs are also insulin-unresponsive [66]. These atypical kinases have a protein binding PB1 domain at their regulatory N-terminus and a PDZ ligand at the C-terminus [64]. Most PKCs, including aPKCs, also have an autoinhibitory pseudosubstrate segment. This pseudosubstrate blocks the active center of the enzyme, and for kinase activation the pseudosubstrate has to be relocated via binding to protein scaffolds, such as PAR6 [67] and p62 [68]. aPKC isoenzymes must be constitutively phosphorylated for their work. Their phosphorylation is independently regulated by two kinases: ribosome-associated mammalian target of rapamycin complex 2 (mTORC2) mediates co-translational phosphorylation of the turn motif, followed by phosphorylation at the activation loop by phosphoinositide-dependent kinase-1 (PDK1). PKCζ has a very low turn-over (about five reactions per minute). It was proposed that scaffolding near substrates may be rate-limiting for PKCζ function [66].

**Figure 2:**
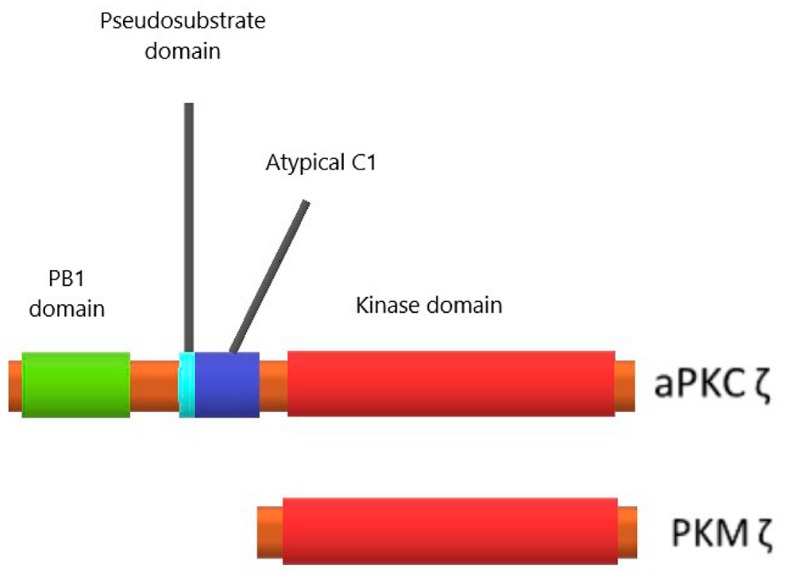
Domain structure of protein kinase Cζ (PKCζ) and its truncated alternative transcript, protein kinase Mζ (PKMζ). In PKCζ, PB1 is the protein-binding domain, pseudosubstrate is a cis-inhibitory element, atypical C1 interacts with diacylglycerol, but it lacks determinants that allow the binding. PKMζ is devoid of all regulatory elements and contains only catalytic domain.

The PKCζ kinase has a shorter alternate transcript, PKMζ, that is expressed exclusively in the central nervous system [69]. This truncated kinase contains only the C-terminal catalytic domain and lacks all N-terminal regulatory domains (PB1, pseudosubstrate and atypical C1) (Figure 2), so it is constitutively active, and because of this its concentration in the cell should be extremely well controlled.

### 3.2. Protein Kinase Mζ Cellular Localization and Function

Newly synthesized PKMζ mRNA is transported to dendrites by the dendrite-targeting element in its untranslated region [70]. Somatodendritic localization of PKMζ in cortical and hippocampal neurons was shown by light and electron microscopy. Immunogold staining revealed PKMζ distribution in spines near or associated with the postsynaptic density, but not in the presynaptic terminal. Also, in some neurons, nuclear localization of PKMζ was reported [71].

Many researchers suggest that PKMζ is a molecule crucial for memory maintenance [72,73,74,75,76,77,78]. 

This idea is based on the following data:

1. Long-term potentiation and memory are accompanied by increasing of the PKMζ protein level in memory-associated structures [79,80,81,82];
2. PKMζ inhibitor ZIP erases established memory in mammals and invertebrates [73,74,75,82,83];
3. PKMζ inhibitor ZIP disrupts the late phase of long-term potentiation, a cellular model of synaptic plasticity and memory [72,81,84];
4. Knockdown of PKMζ in the hippocampus impairs long-term potentiation and negatively regulates memory maintenance [85,86];
5. Overexpression of PKMζ enhances memory [87,88,89].

PKMζ participates in synaptic plasticity control in many ways, as among its substrates are transcription and translation regulators and proteins participating in the rearrangement of postsynaptic density.

Neuronal stimulation induces PKMζ translocation to the nucleus, where it can affect transcription by direct phosphorylation of CBP (CREB-binding protein), that subsequently increases the acetylation level of histones H2B and H3 [90].

Translation regulation by PKMζ may be realized via initiation factors eIF4B and eIF4E. PKMζ directly phosphorylates eIF4B [2]. Phosphorylation of this factor leads to a strong increase in its affinity for non-coding BC1/BC200 RNA. This brain-specific RNA competes with 18S rRNA for translation initiation factors, thereby ensuring a low level of protein synthesis in the neuron [91]. Therefore, phosphorylation of eIF4B by PKMζ suppresses overall translation in neurons. Also, PKMζ phosphorylates Pin1, a peptidyl-prolyl isomerase that may change conformations of initiation factor eIF4E and its regulating proteins and thus suppress translation. Phosphorylation by PKMζ decreases Pin1 binding to translation regulators, promoting protein synthesis [92]. Taken together, these facts let us conclude that PKMζ may be involved in both translation suppression and translation activation mechanisms.

Also, PKMζ participates in the regulation of the function of AMPA glutamate receptors. These receptors are inserted in the postsynaptic membrane during LTP processes, and, on the contrary, removed from the membrane during LTD. PICK1 (protein interacting with C-kinase 1) retains AMPA receptors under the membrane by interaction with the GluA2 subunit of the receptor, preventing its insertion into the postsynaptic membrane. PKMζ disrupts PICK1–GluA2 interaction, after which the GluA2-containing AMPA receptor may be inserted into the membrane [93]. Besides, it was shown that PKM/PKCζ blocker ZIP prevents chemically induced GluA1 and GluA2 phosphorylation, which may indirectly confirm the physiological role of PKMζ in AMPA receptor phosphorylation [94,95]. Moreover, cocaine induces a GluA1 level increase in *nucleus accumbens* after conditioned place preference training, and this effect was prevented by PKMζ inhibitor ZIP [96]. We can suggest that GluA1 increase can be explained by PKMζ-dependent control of translation.

In addition, it was shown that PKMζ regulates phosphorylation of the palmitoylation enzyme zinc finger DHHC-type containing 8 (ZDHHC8), which palmitoylates PSD-95, the major scaffold protein at excitatory synapses. By this, ZDHHC8 promotes PSD-95 insertion to synapse. Inhibition of PKMζ leads to a decrease of synaptic PSD-95 accumulation in vivo, which can be rescued by the overexpression of ZDHHC8 [97].

There are other putative substrates of PKMζ that have yet to be confirmed experimentally. Based on the similarity of PKMζ and PKCζ kinase domains, we speculate that these kinases can phosphorylate the same targets. It was shown that PKCζ activates mTOR signaling, which regulates protein translation and proliferation, in pancreatic β cells and follicular lymphoma [98,99]. It is possible that PKMζ can also regulate mTOR activation in neurons.

### 3.3. Protein Kinase Mζ Translation Regulation: The Role of eIF2α Phosphorylation and Other Possible Mechanisms

PKMζ protein translation in neurons is strongly (but not completely) inhibited. It was demonstrated that PKMζ synthesis depends on many signaling pathways that are involved in LTP (PI3-kinase, CaMKII, MAPK, PKA, mTOR cascades) [81]. Neuronal stimulation causes an increase in the translation of PKMζ in postsynapses, and this increase is necessary for successful LTP formation [76]. The mRNA encoding PKMζ has an extended 5′-UTR. During the splicing process, the 5′-UTR of PKMζ mRNA is modified, and its sequence is unique [69]. Within this 5′-UTR, there are seven short uORFs, which is unusually many. In the work performed in our lab [37], we demonstrated that these uORFs play a critical role in the regulation of PKMζ translation. Elimination of these uORFs by point mutations (AUG to UAG) activated translation of the reporter fluorescent protein in a cell-free system and in primary cultures of rat hippocampal neurons. It seems that all or at least most of seven uORFs contribute to the repression of the main frame translation, because in the succession of genetic constructs, containing 7, 6, 5, 4 or 0 active uORFs in their 5′-UTR, the reporter translation rate increased gradually. We also demonstrated that in cell-free translation systems, translational initiation complexes are formed only on uORFs. Further, we observed an increase in the translation of the reporter protein under the control of wild-type PKMζ 5′-UTR in neuronal culture during non-specific activation by picrotoxin; in cells transfected with a construct containing mutated PKMζ 5′-UTR without uORFs, there was no such PKMζ translation inducibility. Finally, we showed that such a mechanism is similar to the mechanism involved in cell stress response, as application of sodium arsenite to neuron cultures induced translation of mRNA carrying PKMζ 5′-UTR similarly to picrotoxin activation (and again, in cells transfected by a construct without uORFs, arsenite treatment did not change the reporter translation rate). Such similarity of translation shift in neuronal activation and stress response is an indirect indication that phosphorylation of eIF2α may be the main regulator of PKMζ translation. There are literary data supposing that the eIF2α kinase responsible for this regulation may be PERK: in the hippocampal area CA1 of APP/PS1 mice (an animal model of Alzheimer’s disease), PKMζ concentration is reduced, and conditional deletion of PERK restored PKMζ expression back to the normal level [31]. These data may indicate that PERK, unexpectedly, participates in downregulation of PKMζ, but this conclusion would be based on indirect evidence. To determine if PERK actually regulates PKMζ expression, additional experiments with overexpression of PERK in wild-type animals are necessary.

We also demonstrated that for the effective synthesis of 5′-UTR PKMζ, initiation factors eIF4A and eIF4B did not significantly facilitate translation on PKMζ 5′-UTR. However, the truncated form of eIF4G enhanced the efficiency of translation of mRNA with PKMζ 5′-UTR [37]. ΔeIF4G is able to interact with the 40S ribosomal subunit and to bind with mRNA via eIF3, but cannot be involved in the cap-dependent initiation of translation. This form of eIF4G increases the probability of re-initiation events in cell-free systems on uORFs [100]. Activation of translation on the PKMζ 5′-UTR by ΔeIF4G confirms the suggestion on the crucial role of uORFs in translational control of the PKMζ synthesis [37].

PKMζ translation may be additionally regulated via Pin1, since in brain lysates of Pin1^−/−^ mice, both PKCζ and PKMζ concentration were increased, but the exact molecular mechanism of this regulation has not yet been investigated. Considering that, as was mentioned above, PKMζ inhibits Pin1 activity, a feedback with mutual inhibition may be proposed for these two proteins [92]. 

**Figure 3:**
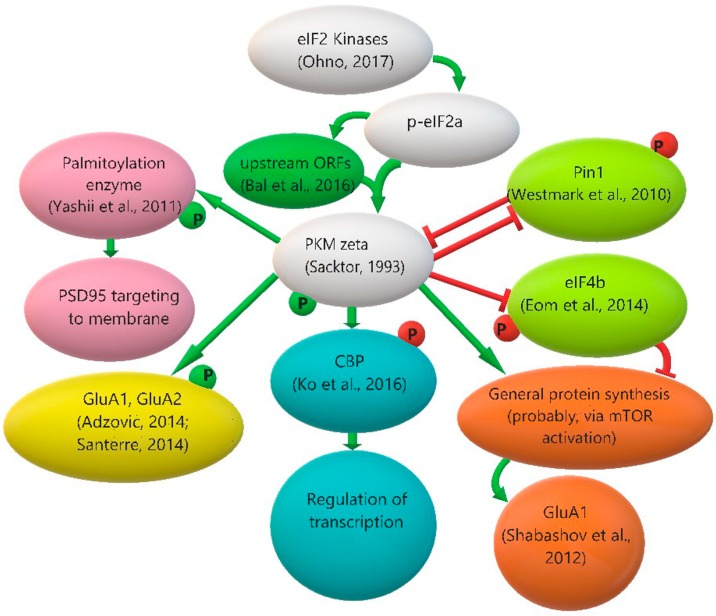
A diagram of PKMζ kinase regulation and functions. eIF2 - eucaryotic translation initiation factor 2; p-eIF2a - phosphorylated α-subunit of eucaryotic translation initiation factor 2; P - phosphate group; ORF - open reading frame; PKM zeta - protein kinase Mζ; Pin1 - peptidyl-prolyl cis-trans isomerase NIMA-interacting 1; PSD95 - postsynaptic density protein 95; eIF4b - eucaryotic initiation factor 4B; CBP - CREB-binding protein; GluA1, GluA2 - glutamate ionotropic receptor AMPA type subunits 1 and 2; mTOR - mammalian target of rapamycin. A detailed explanation of this scheme is presented in Section 3.2 and Section 3.3.

All PKMζ interactions with its substrates and regulators are presented schematically in Figure 3.

## 4. Summary

Phosphorylation of eIF2 α-subunit in neurons leads to a translation shift, suppressing overall translation by a dramatic reduction of the cap-dependent initiation rate while simultaneously increasing the translation of specific mRNAs for which initiation is suppressed in other conditions due to the structure of their 5′-UTRs. It was shown that all four kinases of eIF2α may participate in neuron-specific regulation of these mRNAs; moreover, in many cases, different eIF2α kinases may supposedly be synergic or even interchangeable in their function in neuronal plasticity. p-eIF2α-dependent translation regulation allows the neuron to quickly change protein ensembles at the synapse in an activity-dependent manner, and such regulation is important for both LTP and LTD formation. So, further understanding of eIF2 function in neurons should be an important topic in studying synaptic plasticity. Another essential matter is a potential therapeutic application of researches considering eIF2α phosphorylation. There is a constantly increasing amount of recent data suggesting that eIF2 pathway dysregulation may be an important factor of pathogenesis of many disorders—first of all, Alzheimer’s disease [6,30,31]. So, eIF2 and kinases of its α-subunit, especially PERK and GCN2, may be considered as promising targets for new nootropic medicines. There are also emerging data worth mentioning that p-eIF2α phosphatases, that we did not thoroughly discuss here, may also serve as valid drug targets [101].

## Figures and Tables

**Table 1:** Upstream open reading frames (uORF)-containing mRNAs that code proteins important for neuronal function, and their regulation by kinases of α-subunit of eukaryotic initiation factor 2 (eIF2α).

Protein Name	Protein Function	The Number of uORFs and Presence of Other Translation-Impeding Features in mRNA 5′-UTR	eIF2α Kinases that May be Involved in mRNA Translation Regulation
Activating transcription factor 4 (**ATF4**), also known as cAMP-response element binding protein 2 (**CREB-2**)	transcription repressor	2 uORFs, uORF2 overlaps with the main frame	General control nonderepressible 2 (**GCN2**) [12,31] PKR-like endoplasmic reticulum kinase (**PERK**) [29,31]
Growth arrest and DNA damage-inducible protein (**GADD34**), also known as protein phosphatase regulatory subunit 15A (**PPP1R15A**)	regulatory subunit of p-eIF2α phosphatase, activated by DNA lesion	2 uORFs	**GCN2** and **PERK** [20] *, Protein kinase R (**PKR**) [21] *
CCAAT-enhancer-binding protein homologous protein (**CHOP**), also known as DNA damage-inducible transcript 3 (**DDIT3**)	transcription repressor, activated by DNA lesion	3 uORFs; the short peptide translated from uORF2 is important for the repression of the main frame translation [33]	Presumably *not* **PERK** [31] **GCN2** [22] *
Beta-site APP-cleaving enzyme 1 (**BACE1**)	protease, most known for its role in β-amyloid production	4 uORFs, complicated secondary structure	**PERK** [30] Heme-regulated eIF2α kinase (**HRI**) [14]
Glutamate ionotropic receptor NMDA type subunit 2B (**GluN2B**)	ionotropic glutamate receptor subunit	3 uORFs	**HRI** [15]
Oligophrenin-1	Rho-GTPase-activating protein	2 uORFs	**PKR** [28]
Synapse-Associated Protein 90/Postsynaptic Density Protein-95-Associated Protein 3 (**SAPAP3**)	scaffolding protein of the postsynaptic density	4 uORFs, high GC content; uORF2 is also important for translation start shift between 2 distinct start codons in protein-coding frame [34]	No data
SH3 and multiple ankyrin repeat domains 1 (**Shank1**)	scaffolding protein of the postsynaptic density	3 conventional uORFs (uORF3 overlaps with the main frame) and the fourth unique uORF that starts with non-canonical start codon ACG and upregulates main frame translation	eIF2α phosphorylation is not important for regulation of this mRNA translation [35]
Neuron-specific BCL2-antagonist/killer (**N-Bak**)	pro-apoptotic factor	1 or 2 uORFs in different species; also, 3′-UTR of N-Bak contains premature termination codon and exon–exon junction	eIF2α phosphorylation is not important for regulation of this mRNA translation; it was demonstrated that N-Bak mRNA translation is repressed consistently, even during apoptosis [36]
Protein kinase Mζ (**PKMζ**)	kinase, known for its importance in memory formation	7 uORFs (unusually many, supposedly each of them contributes to the translation repression) [37]	**PERK** [31]

* These data were obtained in experiments addressing cell stress and performed on various non-neuronal cells.

## Abbreviations

∆eIF4G	Truncated eucaryotic translation initiation factor 4G
3′-UTR	3′-Untranslated region
5′-UTR	5′-Untranslated region
7-NI	7-Nitroindazole
AMPA	α-Amino-3-hydroxy-5-methyl-4-isoxazolepropionic acid
aPKC	Atypical protein kinase C
APP	Amyloid precursor protein
ATF4	Activating transcription factor 4
ATF5	Activating transcription factor 5
ATF6	Activating transcription factor 6
ATP	Adenosine triphosphate
BACE1	Beta-site APP-cleaving enzyme 1
BACE1-293	HEK-293 cells stably overexpressing BACE1
Bax	BCL-2-associated X protein
BC1/BC200 RNA	Brain cytoplasmic RNA
BDNF	Brain-derived neurotropic factor
BiP/GPR78	Binding immunoglobulin protein (BiP), also known as 78 kDa glucose-regulated protein
C1	Protein kinase C conserved region 1
C2	Protein kinase C conserved region 2
CA1	Cornu Ammonis region 1
CaMKII	Ca^2+^/calmodulin-dependent protein kinase II
CBP	CREB-binding protein
CHO-K1	Chinese hamster ovary K1 cells
CHOP	CCAAT-enhancer-binding protein homologous protein
CREB-2	cAMP-response element binding protein 2
C-terminal	Carboxyl terminal
DAG	Diacylglycerol
DDIT3	DNA damage-inducible transcript 3
DHPG	Dihydroxyphenylglycine
dsRBM	Double-stranded RNA binding motif
ds-RNA	Double-stranded RNA
eIF2	Eucaryotic translation initiation factor 2
eIF2α	α-subunit of eucaryotic translation initiation factor 2
eIF3	Eucaryotic translation initiation factor 3
eIF4B	Eucaryotic translation initiation factor 4B
eIF4E	Eucaryotic translation initiation factor 4E
eIF4G	Eucaryotic translation initiation factor 4G
ER	Endoplasmic reticulum
G-actin	Globular actin
GADD34	Growth arrest and DNA damage-inducible protein
GCN1	General control of amino-acid synthesis 1-like protein 1
GCN2	General control nonderepressible 2 (kinase)
GCN2DN	Dominant-negative GCN2 allele
GFP	Green fluorescent protein
GluA1	Glutamate ionotropic receptor AMPA type subunit 1
GluA2	Glutamate ionotropic receptor AMPA type subunit 2
GluN2B	Glutamate ionotropic receptor NMDA type subunit 2B
GTP	Guanosine triphosphate
H2B	Histone 2B
H3	Histone 3
HEK	Human embryonic kidney cells
HeLa	Henrietta Lacks cells
HisRS	Histidyl-tRNA synthetase
HRI	Heme-reguated eIF2α kinase
IRE1	Inositol-requiring enzyme 1
ISRIB	Integrated stress response inhibitor
L-LTP	Late long-term potentiation
LTD	Long-term depression
LTP	Long-term potentiation
MAPK	Mitogen-activated protein kinase
Met-tRNAi	Initiator methionyl-tRNA
mGluR	Metabotropic glutamate receptor
mGluR1	Metabotropic glutamate receptor 1
mGluR5	Metabotropic glutamate receptor 5
mGluR-LTD	mGluR-Dependent long-term depression
mRNA	Messenger RNA
mTOR	Mammalian target of rapamycin
mTORC2	Mammalian target of rapamycin complex 2
N-Bak	Neuron-specific BCL2-antagonist/killer
NF-κB	Nuclear factor kappa-light-chain-enhancer of activated B cells
NMDA	*N*-Methyl-d-aspartate
NMDAR	*N*-Methyl-d-aspartate receptor
NMDAR-LTD	NMDAR-dependent long-term depression
nNOS	Neuronal NO-synthase
nt	Nucleotide
N-terminal	Amino-terminal
Ophn1	Oligophrenin-1
P58IPK	Protein kinase inhibitor P58
PACT	Protein activator
PAR6	Partitioning-defective 6
PB1	Phox and Bem1
PDK1	Phosphoinositide-dependent kinase-1
p-eIF2α	Phosphorylated α-subunit of eucaryotic translation initiation factor 2
PERK	Protein kinase R-like endoplasmic reticulum kinase
PERKDN	Dominant-negative PERK allele
PI3-kinase	Phosphoinositide 3-kinase
PICK1	Protein interacting with C-kinase 1
Pin1	Peptidyl-prolyl cis-trans isomerase NIMA-interacting 1
PKA	Protein kinase A
PKC	Protein kinase C
PKCζ	Protein kinase Cζ
PKMζ	Protein kinase Mζ
PKR	Protein kinase R
PP1c	Protein phosphatase 1c
PPP1R15A	Protein phosphatase regulatory subunit 15A
PS1	Presenilin 1
PSD	Postsynaptic density
PSD-95	Postsynaptic density protein 95
Rho	Ras homolog
RNA-seq	RNA sequencing
rRNA	Ribosomal RNA
RT-qPCR	Reverse transcription quantitative polymerase chain reaction
RWD	Domain in RING finger and WD repeat containing proteins and DEXDc-like helicases
Sal003	Salubrinal analog 003
SAPAP3	Synapse-associated protein 90/postsynaptic density protein-95-associated protein 3
Shank1	SH3 and multiple ankyrin repeat domains 1
shRNA	Small hairpin RNA
siHRI	Small interfering RNA that binds with HRI mRNA
siRNA	Small interfering RNA
SNP	Sodium nitroprusside
SS	Signal sequence
STAT	Signal transducer and activator of transcription
Tg2576	AβPP-transgenic 2576 mice
TM	Transmembrane segment
tRNA	Transfer RNA
uORF	Upstream open reading frame
UPR	Unfolded protein response
UV	Ultraviolet
VEGF-A	Vascular endothelial growth factor A
XBP-1	X-box binding protein 1
ZDHHC8	Zinc finger DHHC-type containing 8
ZIP	Zeta inhibitory peptide
